# The Effect of Information on the Time Course of Pain During an Episode of Acute Experimentally Induced Low Back Pain—A Randomised Experiment

**DOI:** 10.1002/ejp.70011

**Published:** 2025-04-20

**Authors:** M. Travers, B. M. Wand, D. Hince, W. Gibson, S. Meldgaard Hansen, T. Sigurðsson, S. Sorensen, T. Skuli Palsson

**Affiliations:** ^1^ School of Health Sciences The University of Notre Dame Fremantle Australia; ^2^ Institute of Health Research The University of Notre Dame Australia; ^3^ Department of Clinical Medicine Aalborg University Aalborg Denmark; ^4^ Greenland Healthcare Nuuk Greenland; ^5^ Department of Physiotherapy and Occupational Therapy Aalborg University Hospital Aalborg Denmark

**Keywords:** diagnosis, low back pain, musculoskeletal pain, pain perception, randomised controlled experiment

## Abstract

**Background:**

We compared the time course of pain intensity ratings between two groups who were given different information during an episode of acute experimentally induced LBP.

**Methods:**

Fifty weight‐training naive and pain‐free people participated in this randomised clinical experiment. Immediately after performing a back exercise intended to cause delayed onset muscle soreness, one group was told that their muscles had been damaged and advised they needed to protect their back over the coming days. The other group's symptoms were described in terms of tissue sensitisation, and they were advised to keep moving.

The primary outcome was movement‐evoked low back pain intensity measured using an 11‐point numeric rating scale (NRS 0–10). Pain intensity was recorded at baseline, immediately after the intervention and then daily for 7 days. The method of generalised estimating equations (GEE) was used to estimate treatment effects for average daily pain.

**Results:**

Movement‐evoked pain intensity scores changed over time in both groups (main effect of time: χ^2(7) = 246.2, *p* < 0.001). However, the intervention did not affect movement‐evoked pain intensity scores (main effect of group: χ^2(1) = 0.02, *p* < 0.895). The adjusted mean difference between the groups was only −0.05/10 (95% CI –0.72 to 0.63, *p* = 0.895) when averaged across all time points.

**Conclusions:**

We simulated an episode of low back pain and found that information based on tissue sensitivity and advice to remain active did not improve pain compared to information referencing tissue damage and advice to rest and protect the back.

**Significance Statement:**

Contemporary clinical guidelines and models of care recommend avoiding pathoanatomical diagnostic labels and encourage clinicians to advise patients to stay active during an episode of acute low back pain (LBP).

We simulated an episode of acute LBP and found that information based on tissue sensitivity and advice to remain active did not improve pain compared to information referencing tissue damage and advice to rest and protect the back. The results could be different if repeated in a clinical population.

## 1 Introduction

Acute low back pain (LBP) is a common occurrence. The 1‐year incidence of a first‐ever episode of LBP is estimated to be 6.3%–15.4%, and the 1‐year incidence of people who have any episode of LBP (first‐ever or recurrent) is estimated to be 1.5%–36% (Hoy et al. 2010). Multiple guidelines recommend that clinicians advise patients to stay active during an episode of acute LBP (Maher et al. 2023; O'Connell et al. 2016; Oliveira et al. 2018). In fact, clinicians are explicitly advised to explain to patients that remaining active will expedite recovery (Apeldoorn et al. 2024). Contemporary models of care for LBP also emphasise avoiding the use of patho‐anatomical diagnostic labels and descriptors that cannot be confirmed by clinical testing (Bernstein et al. 2017; Palsson et al. 2019; Stochkendahl et al. 2017). Indeed, recent guidelines of the Royal Dutch Society for Physical Therapy recommended that clinicians avoid language that might cause catastrophic thinking and fear of pain such as ‘injury’, ‘degeneration’ and ‘wear and tear’ (Apeldoorn et al. 2024). However, it is unknown whether receiving nonpathoanatomical information regarding the cause of LBP and advice to stay active have a positive effect on pain trajectory during an episode of acute LBP.

This study aimed to compare the time course of pain intensity ratings between a group that was specifically told that they had been injured and advised they needed to protect their back and a group whose symptoms were described in terms of tissue sensitisation and advised to keep moving during an acute episode of experimentally induced LBP.

## 2 Methods

### 2.1 Study Design

This was a prospectively registered (ACTRN12618000042246), parallel, two‐arm, randomised clinical experiment with concealed allocation and intention‐to‐treat analyses. A random number sequence was computer‐generated by a researcher not involved with recruitment, and concealment was ensured by sealing allocations in opaque, consecutively numbered envelopes. To facilitate masking, all participants were told the study was investigating the effect of different types of information on pain, but they were not informed of the nature of the information for each group nor the hypothesis of the study. Participants were only made aware of these details after data collection was complete. All outcome measures were self‐reported; therefore, all outcomes were recorded by assessors masked to study hypotheses. Furthermore, all analyses were undertaken by a statistician (DH) masked to group allocation. All participants provided written consent before they enrolled in this study. Ethics approval was granted by the ethical committee of Northern Denmark (N‐20150048). Participants attended an initial session at Aalborg University for the collection of baseline data; all follow‐up data were self‐reported online.

### 2.2 Participants

Recruitment occurred between February and April 2022. Participants were recruited by an advertisement at the University campus and word of mouth. We included pain‐free, weight‐training naive people aged 18–40 years who could understand and read Danish or English. Participants were excluded if they: had a current musculoskeletal pain problem; had lower limb pain or low back pain that required a visit to a health care professional within the previous 12 months; had sustained a traumatic injury (e.g., fracture or dislocation) of the lower limb or spine within the previous 5 years; had any history of persistent (> 3 months) lumbopelvic pain; reported any ongoing significant medical conditions; consumed regular anticoagulant medication or medications known to influence pain sensitivity (e.g., painkillers, anti‐inflammatories or antidepressants); had participated in studies using a similar experimental pain model; or had recently trained the low back or legs with resistance exercises.

### 2.3 Procedures

#### 2.3.1 Day 0

Potential participants responded to advertisements on social media and were placed at various locations on the Aalborg University campus. After a preliminary screening by phone or email, potential participants were invited to attend Aalborg University for possible inclusion in the study. Inclusion criteria were rechecked, and eligible participants were provided with study information and afforded the opportunity to ask questions about the study. Willing participants signed a consent form and provided demographic details (gender, age, height, and weight). Participants were then randomly assigned to a group by opening the next numbered envelope, at which point they were considered enrolled. Group allocation was performed by Researcher 1 (SMH) who did not disclose the assignment to Researcher 2 (TS) at any point during the study.

After randomisation, Researcher 2 oversaw the assessment of the baseline outcome measures. Participants were invited to complete the Tampa Scale of Kinesiophobia (TSK‐17) and the Pain Catastrophising Scale (PCS) and pain intensity during maximal forward bending was recorded for each participant. Next, participants undertook the delayed onset muscle soreness protocol (see below) in the presence of Researcher 2.

Upon completion of the exercise protocol, participants were then taken to an adjoining room where the assigned intervention was delivered by Researcher 1. Participants then returned to the first room where all outcome measures were reassessed by Researcher 2. Participants were instructed not to discuss any aspect of the intervention they received from Researcher 2 to maintain masking.

#### 2.3.2 Days 1–7

Over the following 7 days, a daily online questionnaire was sent to the participants using REDCap (*version 10.0.23*), which they were instructed to complete at the end of each day. The questionnaire asked them to record their pain intensity with forward bending and average pain intensity with daily activity during that day. The level of kinesiophobia and pain‐related catastrophising was also assessed using the same questionnaires employed at baseline.

#### 2.3.3 Day 8

On Day 8, participants were contacted by phone by Researcher 1 to assess for any adverse effects, ensure that all pain had subsided and disclose the nature of the study. No outcome data were collected on this day.

### 2.4 Delayed Onset Muscle Soreness Protocol

We used a previously established protocol to induce delayed onset muscle soreness (DOMS) in the lumbar spine extensors (Bishop et al. 2011a, 2011b). Participants lay prone, with their pelvis on the edge of a plinth and their legs fixed with straps. Participants were instructed to cross the arms in front of the chest and lower the upper half of their body towards the floor, flexing fully at both the hips and lumbar spine and then return to the horizontal position. Each repetition was performed through the full available range of motion, and participants were encouraged to choose their own pace until momentary failure. Momentary failure is defined as ending the working set when the participant reaches the point where, despite attempting to do so, they cannot complete the concentric portion of their current repetition without deviation from the prescribed form of the exercise (Steele et al. 2017). Participants performed four sets to momentary failure with an interset rest period of 60 s.

### 2.5 Interventions

#### 2.5.1 Experimental Group: Education and Advice Based on a Temporary Sensitisation Model (TSM)

In this group, there was no reference to tissue injury as a contributing factor to their back pain. Participants were informed that the exercise protocol would result in a short‐term overload of the back muscles and that this would likely cause their muscles to become sensitive as a normal result of doing an unaccustomed exercise. Moreover, they were told that this was normal for the muscle and in fact a necessary part of the back muscles becoming stronger. They were informed that the symptoms were not due to tissue injury but as a result of short‐term changes in the concentration of some chemicals in the back muscles. They were advised to stay active and move as usual to aid blood flow and help remove the chemicals that made the back sore. We told them that if they protected their back from load and movement, it could mean that their back would remain sore for a longer time. Participants were advised that they did not have to change their behaviour in terms of work and daily activities, but it was recommended that they minimise the amount of heavy lifting or very strenuous exercise during the next 3 days.

#### 2.5.2 Control Group: Education and Advice Based on an Injury Model (IM)

In this group, there was explicit reference to tissue injury as a contributing factor to their back pain. Participants were informed that the exercise protocol would result in a small amount of tearing of muscle fibres within the back, similar to the fraying of a rope under load. They were told that the symptoms they were experiencing were a result of this muscle damage and accompanying bleeding and swelling in the back muscles. Participants were advised to avoid engaging in strenuous activity with their backs to avoid further damage in the first few days. They were given an example of when we sprain our ankle, it is normal to limp and temporarily limit the movement and load. Participants were told that pain should be their guide for the following few days in terms of their activity level and how much they moved their back. To help speed up the healing process, it was recommended they avoid activities that could increase the symptoms they experienced. Likewise, they were recommended to be more careful with their back when performing their usual work and daily activities (e.g., sit up straight and lift with a straight back).

The same researcher delivered all educational sessions to both groups and was therefore unmasked. However, to minimise performance bias, a standardised script was developed to ensure consistency in messaging (see Supporting Information).

### 2.6 Primary Outcome

The primary outcome was movement‐evoked low back pain intensity. Participants were instructed to stand with their feet shoulder‐width apart, to keep their legs straight, lean forwards to try and touch their toes and note the maximal pain they experienced during the task. Immediately upon completion of the task, maximal pain intensity was recorded using an 11‐point numeric rating scale (NRS 0–10), where 0 was anchored with no pain and 10 was defined as the worst pain imaginable (Dworkin et al. 2005). Pain intensity was recorded at baseline, immediately after the intervention and then daily for 7 days. To collect the daily outcome measures, participants were instructed to perform the forward bending test and record the pain intensity they experienced at the end of the day, just before bedtime.

### 2.7 Secondary Outcomes

Daily activity‐related average pain intensity was reported using an 11‐point (0–10) NRS. Participants were asked to indicate the average pain intensity they experienced in their back while performing their normal activities that day. For this outcome, 0 was anchored with no pain and 10 was defined as the worst pain imaginable (Dworkin et al. 2005). This outcome was collected at the end of each day from Day 1 to Day 7.

Kinesiophobia was measured using the Tampa Scale of Kinesiophobia (TSK) (range, 17–68; higher scores are worse; minimally clinically important difference (MCID) not established) (Houben et al. 2005). The Pain Catastrophising Scale (PCS) was used to explore pain catastrophising (range, 0–52; higher scores are worse; MCID not established) (Sullivan et al. 1995).

### 2.8 Sample Size

A power analysis using G*Power 3.1.9.2 (*Heinrich Heine University, Dusseldorf Germany*) was used to estimate the sample size. With the significance level set at *p* < 0.05, a 0.5 correlation among repeated measures and the effect size for the interaction between time and treatment assumed to be small (0.2), it was determined that a total of 20 per group (two groups) would yield a power of 0.90 for five repeated measurements. To account for potential dropouts, 25 subjects were recruited in each group. The decision to control for baseline outcome levels and the inclusion of eight repeated measures further increased the estimated power for this test.

### 2.9 Statistical Analysis

All data were analysed using Stata v17 (*StataCorp LLC, College Station, TX, USA*). Data were summarised using means/standard deviations (SDs) for continuous variables and frequencies/percentages for categorical variables. *p* < 0.05 was considered evidence for a difference with all inferential tests described below.

Comparison between those who were/were not lost to follow‐up was made using independent t‐tests and χ^2^ tests. The effects of group (temporary sensitisation model (TSM) versus injury model (IM)) on primary (movement‐evoked pain intensity) and secondary (PCS, TSK and average daily pain intensity) outcomes immediately postintervention and over the 7‐day follow‐up period were assessed after adjusting for the relevant ‘baseline’ variable measured prior to the intervention. For movement‐evoked pain intensity, the ‘baseline’ values were recorded immediately after the DOMS protocol. PCS and TSK ‘baseline’ values were recorded prior to the DOMS protocol, and there was no assessment of average daily pain until the day after the intervention. For all outcomes, a preliminary model was fitted including the group (TSM vs. IM) by day (1–7 as a categorical variable) interaction. If the Wald χ^2^ test for the interaction returned a *p* < 0.05, the effect of group was interpreted from this interaction model; otherwise, only the main effects of group, day and the relevant ‘baseline’ variable were included in the model used to estimate the effect of the intervention.

The models were all fitted using a generalised linear regression model (GLMM) appropriate for the distribution of the outcome variable, with the factors described above included as fixed effects. Random effects were also included to account for the correlation structure. Although mixed effect models were specified in the trial protocol, the method of generalised estimating equations was used to estimate treatment effects for average daily pain because the GLMM failed to converge. Specifications for all models used are presented in Table S1.

All outcomes are presented as adjusted means, adjusted mean differences and accompanying 95% CIs. Main effects and interactions were assessed using approximate Wald χ^2^ tests. The observed (unadjusted) means and standard deviations for all outcomes are presented in Table 2 for comparison. The beta coefficients, 95% CIs and *p*‐values estimated by all models reported are displayed in Table S2.

## 3 Results

### 3.1 Compliance With Trial Protocol

Our recruitment target was achieved, and all enrolled participants met eligibility criteria. All participants completed the DOMS protocol and received the intervention to which they were allocated. Analyses were conducted as per the registered protocol with the addition of adjusting for ‘baseline’ values in the models. One of the outcome measures outlined in the registered protocol, pressure–pain thresholds, was part of a nested study and thus will be reported in a separate manuscript.

### 3.2 Participants

**FIGURE 1:**
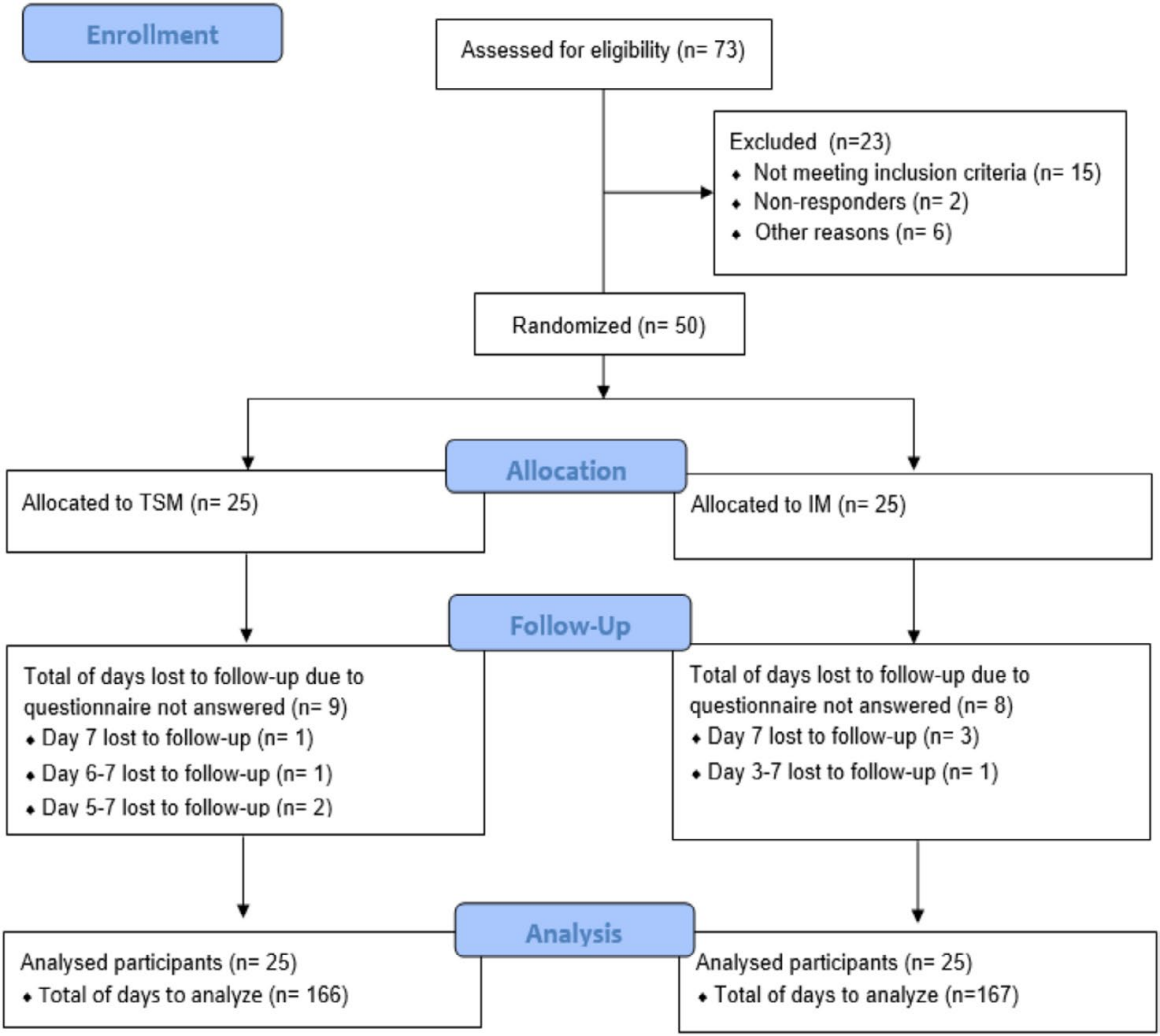
CONSORT diagram of participant progress through the trial. IM: Injury model. TSM: Temporary sensitisation model.

Seventy‐three individuals were assessed for eligibility. Fifty participants were randomly assigned to TSM (*n* = 25) or IM (*n* = 25; Figure 1). All participants completed all tasks between Day 0 and Day 2. One participant (2%) did not complete the primary or secondary outcomes on Day 3 and Day 4, three (6%) on Day 5, four (8%) on Day 6 and eight (16%) on Day 7. This represents a total of 17 participant days lost to follow‐up across the duration of the study or 4.3% of total participant days. Loss to follow‐up was balanced across groups with nine data points missing in the TSM group and eight in the IM group. Participants who did not complete all primary outcome assessments did not differ from those that did in terms of all variables listed in Table 1, except for completing on average 16 more back extensions [mean (SD): 86 (20) vs. 102 (20) extensions, *t*(48) = −2.1, *p* = 0.040].

**TABLE 1:** Demographic and trial variable summaries for TSM and IM groups at baseline.

Variable	TSM mean (SD)	IM mean (SD)	Total mean (SD)
*N*	25	25	50
Female *n* (%)	10 (40)	10 (40)	20 (40)
Age	25.8 (3.3)	26.7 (4.4)	26.2 (3.9)
BMI	25.0 (4.2)	25.3 (4.4)	25.2 (4.1)
Number of back extensions			
Set 1	32.4 (11.0)	30.6 (9.5)	31.5 (10.2)
Set 2	21.4 (6.4)	20.0 (5.5)	20.7 (6.0)
Set 3	18.0 (4.1)	18.0 (4.4)	18.0 (4.2)
Set 4	17.8 (5.1)	19.0 (5.7)	18.4 (5.4)
Total	89.5 (22.2)	87.6 (18.7)	88.5 (20.4)
Movement‐evoked pain	0 (0)	0 (0)	0 (0)

Abbreviations: IM, injury model; TSM, temporary sensitisation model.

Demographics and descriptive data are summarised in Table 1. Participants were generally young [mean (SD): 26.7 (3.9) years] and 40% (*n* = 20) were female. Both groups completed a similar number of back extensions during the DOMS protocol, with an average of just under 90 repetitions across the four sets in both groups. Mean PCS and TSK scores were within the normal range (Table 2). No adverse events were reported.

**TABLE 2:** Observed mean/standard deviation (SD) for TSM and IM groups for primary and secondary outcomes measured at baseline and over the 7‐day follow‐up period.

Outcome	Day	TMS		IM	
		Mean	SD	Mean	SD
Movement‐evoked pain intensity (0–10)^a^	*0*—Post‐DOMS protocol	2.1	1.8	2.3	2.1
	*0*—Postintervention	1.3	1.5	1.1	1.3
	*1*	2.8	1.9	3.4	2.4
	2	3.2	2.7	3.0	2.5
	*3*	1.6	1.7	1.5	1.7
	*4*	0.8	1.1	0.8	1.1
	5	0.6	0.8	0.7	1.5
	6	0.5	1.0	0.3	0.6
	7	0.3	0.7	0.3	0.6
PCS	*0*—Pre‐DOMS protocol	15.4	9.8	14.0	9.1
	*0*—Postintervention	15.6	9.1	15.4	8.5
	*1*	15.4	9.8	14.0	9.1
	2	15.2	10.7	13.5	9.6
	*3*	14.6	10.2	12.1	11.3
	*4*	13.9	10.6	11.7	10.5
	5	13.2	11.0	11.3	10.5
	6	13.3	10.7	10.9	11.3
	7	13.2	10.5	11.5	11.6
TSK	*0‐*Pre‐DOMS protocol	37.8	7.7	32.5	6.1
	*0‐*Postintervention	33.6	6.5	33.9	7.3
	*1*	33.4	6.3	34.1	5.9
	2	32.6	7.1	33.9	6.3
	*3*	32.2	6.1	34.2	6.5
	*4*	33.2	6.4	34.1	6.0
	5	32.1	5.9	33.8	6.3
	6	31.0	6.5	33.6	6.7
	7	31.6	6.5	34.5	6.2
Average daily pain intensity (0–10)	*1*	2.6	1.9	3.1	2.2
	2	3.2	2.6	2.5	2.6
	*3*	1.7	1.8	1.5	1.9
	*4*	1.0	1.1	0.8	1.2
	5	0.5	0.8	0.7	1.5
	6	0.5	0.7	0.4	0.9
	7	0.2	0.4	0.3	0.6

Abbreviations: DOMS, delayed onset muscle soreness; IM, injury model; PCS, Pain Catastrophising Scale; TSK, Tampa Scale of Kinesiophobia; TSM, temporary sensitisation model.

^a^ Denotes primary outcome for the study.

### 3.3 Primary Outcome: Movement‐Evoked Pain Intensity

**FIGURE 2:**
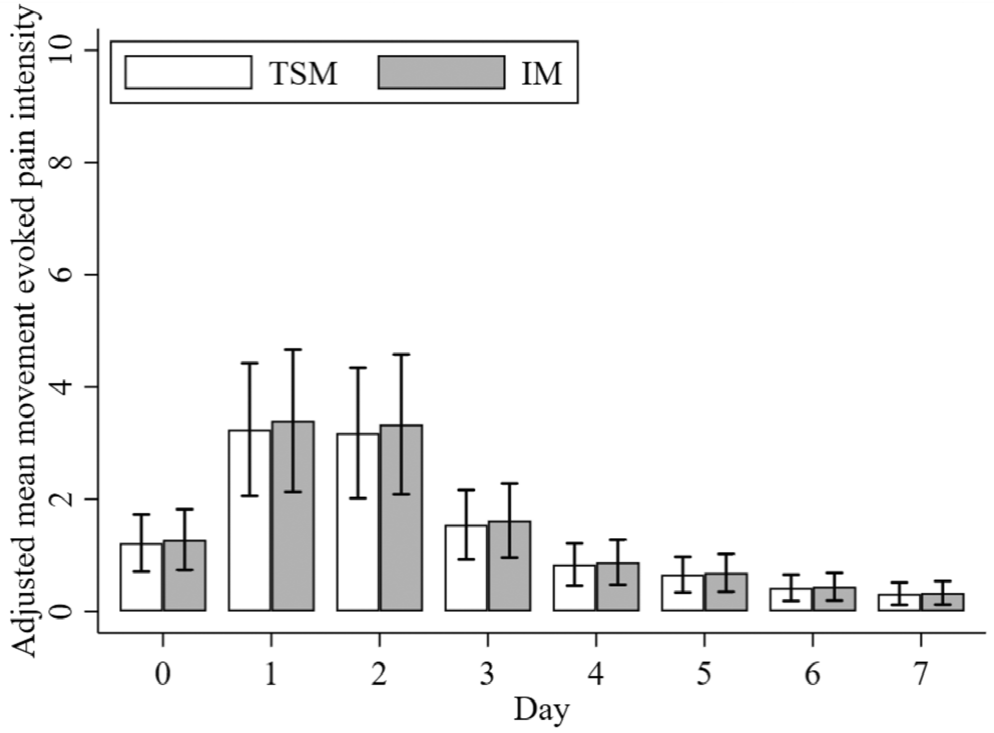
Movement‐evoked pain intensity means for temporary sensitisation model (TSM) and injury model (IM) groups assessed immediately postintervention and over the 7‐day follow‐up period, following adjustment for baseline movement‐evoked pain intensity values. Error bars represent the 95% confidence intervals.

There was no evidence that the change in movement‐evoked pain intensity over time differed between groups [Time × Group interaction: χ^2^(7) = 4.40, *p* = 0.732]. Movement‐evoked pain intensity scores changed over time in both groups [main effect of time: χ^2^(7) = 246.2, *p* < 0.001]. However, the intervention did not affect movement‐evoked pain intensity scores [main effect of group: χ^2^(1) = 0.02, *p* < 0.895]. The adjusted mean difference between the TSM and IM groups was only −0.05/10 (95% CI –0.72 to 0.63, *p* = 0.895, see Figure 2) when averaged across all time points.

### 3.4 Secondary Outcomes: PCS, TSK and Average Daily Pain Intensity

#### 3.4.1 PCS

**FIGURE 3:**
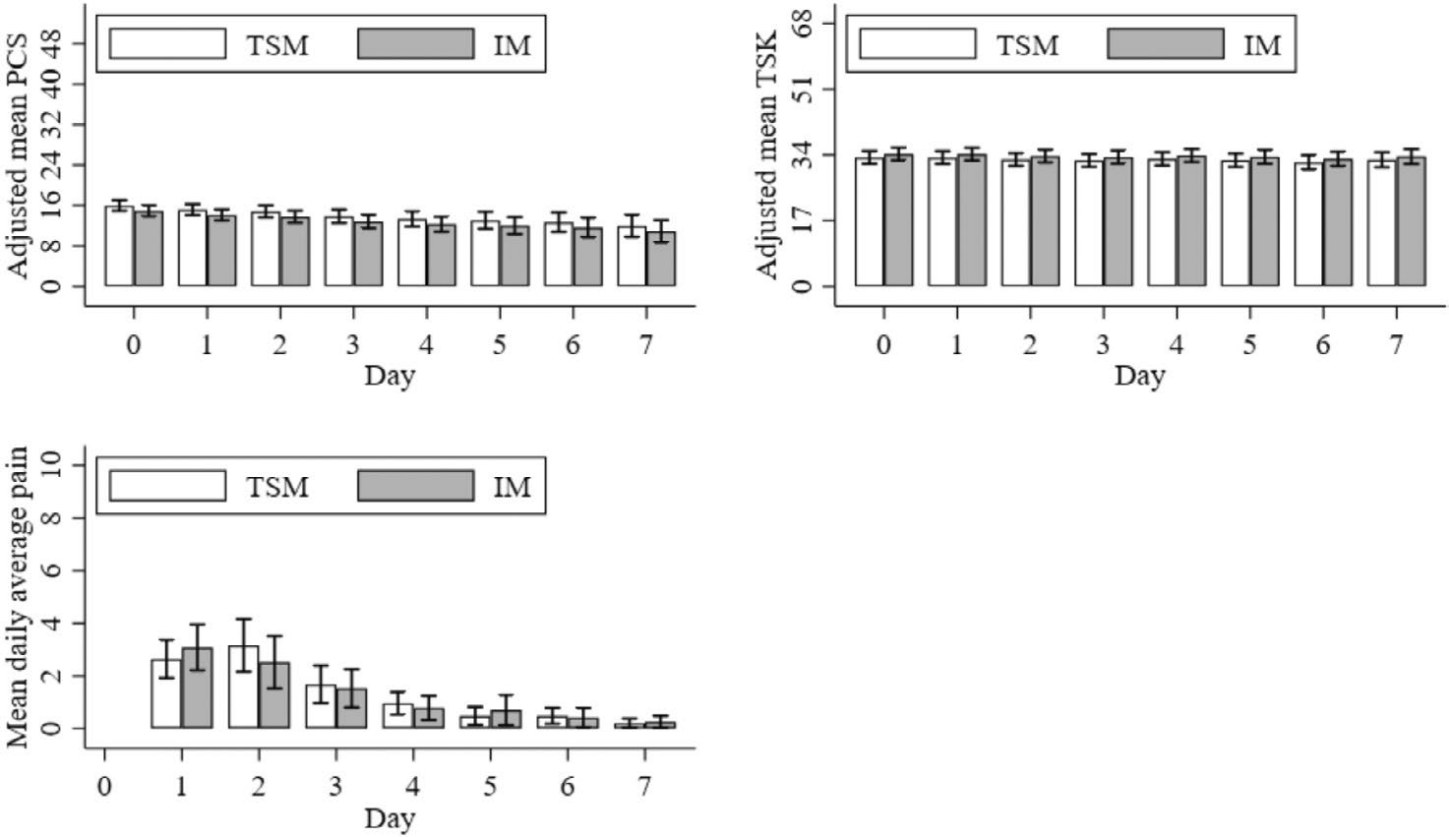
Secondary outcome variable means for temporary sensitisation model (TSM) and injury model (IM) groups assessed over the 7‐day follow‐up period. Means are adjusted for outcome variable ‘baseline’ values measured prior to the intervention (except for average pain intensity, as no measure of this outcome was taken on Day 0). Error bars represent the 95% confidence intervals. PCS: Pain Catastrophising Scale; TSK: Tampa Scale for Kinesiophobia.

Pain catastrophising consistently reduced over the 7‐day follow‐up in both groups [Figure 3 top left; effect of Day χ^2^(7) = 14.7, *p* = 0.039], with a reduction between Day 0 and Day 7 of −4.0 points (95% CI –6.3 to –1.8, *p* < 0.001). However, adjusted PCS means did not differ between groups [main effect of group: χ^2^(1) = 0.03, *p* = 0.852, adjusted mean difference 0.1, 95% CI –1.4 to 0.4], nor was there evidence for the Time × Group interaction [χ^2^(7) = 2.8, *p* = 0.906].

#### 3.4.2 TSK

There was no evidence of any change in kinesiophobia across the course of the study main effect of time: χ^2^ (7) = 8.3, *p* = 0.307]. However, the TSM group recorded consistently lower TSK scores compared with the IM group [main effect of group: χ^2^(1) = 13.1, *p* < 0.001, adjusted mean difference: 4.4, 95% CI 2.0–6.8; Figure 3 top right]. No evidence for a Time × Group interaction on TSK was noted [χ^2^(7) = 7.3, *p* = 0.398].

#### 3.4.3 Average Daily Pain Intensity

The effect of the intervention on adjusted mean average daily pain intensity scores depended upon the time postintervention [group‐by‐day interaction: χ^2^(6) = 16.6, *p* = 0.011]. The net change in average pain between the intervention groups from Days 1 to 2 was −1.1 (95% CI –2.0 to −0.2, *p* = 0.020). This was the combined effect of an *increase* from Days 1 to 2 in the TSM group (adjusted mean difference: 0.5, 95% CI –0.1 to 1.1, *p* = 0.097), and a *decrease* in the IM group (adjusted mean difference: –0.6, 95% CI –1.2 to 0.11, *p* = 0.103). From Day 3, however, daily pain intensity decreased with each consecutive day to a similar extent in both groups, and there was no evidence for any further group differences at these later time points (see Figure 3, bottom left).

## 4 Discussion

The aim of this study was to investigate if the diagnostic information and associated advice on activity levels dispensed by a clinician affected pain outcomes in people with acute experimental low back pain. To address this aim, we employed a parallel, two‐arm, randomised clinical experiment with concealed allocation and intention‐to‐treat analyses. We induced DOMS in the back muscles of a group of healthy volunteers and randomly assigned participants to receive either education and advice based on a temporary sensitisation model or education and advice based on a tissue injury model. We hypothesised that the group receiving the temporary sensitisation explanation and advice to stay active would have a more favourable pain trajectory than those given an injury‐based explanation and advised to rest and protect the back. We found no difference in our primary outcome of movement‐evoked pain intensity between the two groups at any time over the 7‐day test period. Similarly, we observed no differences in the secondary outcomes of average daily pain intensity or pain catastrophising. We did note a between‐group difference for kinesiophobia favouring the TSM group immediately postintervention and the TSM recorded consistently lower kinesiophobia scores compared with the IM group across the duration of the study. However, we urge caution in the interpretation of the observed difference as the meaningfulness of increased kinesiophobia in our population is questionable in the absence of evidence of a difference in pain intensity between groups.

The notion that the information given to a person about the state of their back could influence their pain trajectory is not unfounded. Previous experiments have demonstrated that subjective experiences associated with LBP can be immediately modulated by various forms of information that might impact how people view their back. Seeing the back can be analgesic during the application of massage to the back (Loffler et al. 2017) and indeed even at rest, for people with chronic LBP (Diers et al. 2016). Furthermore, visualising the back while bending seems to reduce movement‐evoked pain in people whose back pain is usually evoked by bending (Wand et al. 2012). Even manipulating auditory feedback during the application of force to the spine alters participants' sense of stiffness in the back (Stanton et al. 2017). Few studies have tested specifically if diagnostic information has a direct effect on LBP. Rajasekaran et al. (2021) tested the effect of providing pathoanatomical information from an MRI report versus reassuring information regarding MRI findings in people with chronic LBP. In contrast to our finding, participants who received a full factual explanation of the pathologies observed on their MRI scan reported substantially higher pain intensity after 6 weeks compared to people with chronic LBP who were reassured that their scan represented ‘normal age‐related changes’ (Rajasekaran et al. 2021). There are some key differences between our experiment and the trial by Rajasekaran et al. (2021); we were interested in the daily trajectory of pain in an acute episode of experimentally induced LBP whereas they recruited people with chronic LBP and evaluated outcome over a longer time period.

Inducing DOMS in the back muscles is an established method for studying various aspects of the LBP experience (Bishop et al. 2011a, 2011b), though there are clear differences in the clinical experience of acute LBP. We recruited weight‐training‐naive participants to maximise the potential threat value and unfamiliarity of DOMS in the back area. However, it is possible that the threat value of DOMS was not sufficient for our interventions to have an effect, and the result could be different if this experiment was performed in a clinical population. In support of this idea, though not in the back, a recent randomised controlled experiment has demonstrated that diagnostic information has an immediate effect on Achilles tendon pain during hopping in people with Achilles tendinopathy (Travers et al. In Press).

Psychological features of pain are thought to be important factors in shaping the recovery trajectory (Corrêa et al. 2022; Jackson et al. 2014; Matsudaira et al. 2014; Trinderup et al. 2018; Wertli et al. 2014). Accordingly, our secondary outcomes included fear of movement and pain catastrophising. As stated above, we observed no meaningful differences between groups with respect to pain catastrophising, though fear of movement was slightly lower in those receiving the TSM‐aligned advice. The clinical significance of this is unclear as the difference is small and there was no associated improvement in pain‐related outcomes. However, there are data demonstrating that fear of movement is causally related to outcomes in clinical presentations of acute low back pain (Traeger et al. 2019), so this might be an important finding if replicated in the context of clinical pain. Further research in clinical populations would clarify this relationship further.

All studies carry limitations that should be considered when interpreting their results. In the present study, the participants were masked to the study hypothesis and the nature of the intervention received, and the researcher delivering the intervention used a standardised script. As such, while we endeavoured to minimise performance and detection biases, we cannot be certain that our measures were adequate in this regard. To ensure that the setting was as authentic as possible, the study was conducted in a clinical environment, and the experimental procedures were run by registered physiotherapists. However, the fact that recruitment and the intervention were both delivered by registered physiotherapists may have reduced the threat value of the intervention in the present study. Finally, we used DOMS as a model for acute LBP. Therefore, the fact that we observed no difference in the trajectory of pain between the two groups does not necessarily mean that contemporary models of care and the recommendations of LBP guidelines are incorrect. It may be that experimentally induced back pain is not suitable for detecting such an effect. The results of this experiment could be different if performed on a clinical population experiencing an acute episode of LBP and is a natural next step in this line of research. As yet, we do not know if the diagnostic information delivered by a clinician during an acute episode of LBP influences the pain trajectory.

## 5 Conclusion

Best practice guidelines and current models of care recommend clinicians avoid unfounded pathoanatomical diagnostic information and encourage maintaining activity during an episode of acute low back pain. We simulated an episode of low back pain and found that information based on tissue sensitivity and advice to remain active did not improve pain compared to information referencing tissue damage and advice to rest and protect the back. The results could be different if repeated in a clinical population.

## Author Contributions

This study was designed by M.T., B.M.W., D.H., W.G. and T.S.P. The experiments were performed by T.S., S.M.H., S.S. and T.S.P. The data were analysed by D.H., and the results were critically examined by all authors. M.T. and B.M.W. had a primary role in preparing the manuscript, which was edited by all authors. All authors have approved the final version of the manuscript and agree to be accountable for all aspects of the work.

## Conflicts of Interest

M.T. has received speaker fees for presentations and lectures on pain and exercise rehabilitation. B.M.W. has received speaker fees for presentations and lectures on pain and exercise rehabilitation. The remaining authors have nothing to declare.

## Plagiarism Declaration

We hereby declare that this paper is our own work, except where acknowledged and has not been submitted elsewhere.

## Public Data Depository

If you have a public depository for your data, please give the DOI here.

## Reviewer List

To avoid conflicts of interest, please suggest reviewers from different countries and with different nationalities. Please note that it is the authors' responsibility to suggest appropriate reviewers. The failure to do so signifies that your work will not draw broad attention from readers and is susceptible to rejection.

## Observational Study

The authors have nothing to report.

## Systematic Review

The authors have nothing to report.

## Animal Study

The authors have nothing to report.

## Use of Artificial Intelligence

AI was not used in any aspect of the generation of this manuscript.

## Note

Manuscripts not complying with the authors’ guideline will be returned without processing. https://onlinelibrary.wiley.com/page/journal/15322149/homepage/forauthors.html.

## Supporting information


Data S1.
