# Supplementary material for: The Effect of Information on the Time Course of Pain During an Episode of Acute Experimentally Induced Low Back Pain—A Randomised Experiment
Source: Eur J Pain. 2025 Apr 20;29(5):e70011. doi: 10.1002/ejp.70011 (PMC12010030; doi:10.1002/ejp.70011)
Supplement: Supplementary file 1 — Data S1. [file EJP-29-0-s001.docx]

Supplementary Table 1. Regression models fitted to the primary and secondary outcomes.

| Outcome | Distribution | Link Function | Method for managing repeated measures |
| --- | --- | --- | --- |
| Movement evoked pain intensity | Negative binomial | Natural log | Random intercept model |
| PCS | Normal | Identity | Random slope model |
| TSK | Normal | Identity | Random slope model |
| Average daily pain intensity | Negative binomial | Natural log | GEE using exchangeable correlation structure with robust standard errors |

PCS: Pain Catastrophising Scale; TSK: Tampa Scale of Kinesiophobia; GEE: generalised estimating equations used for estimation.

Supplementary Table 2. Beta coefficients (*β*), 95% confidence intervals (95% CI) and *p*-values from the regression models fitted to the primary and secondary outcomes.

|  | Movement evoked pain intensity | | | PCS | | | TSK | | | Daily Average Pain | | |
| --- | --- | --- | --- | --- | --- | --- | --- | --- | --- | --- | --- | --- |
|  | *Exp(β)* | 95% CI | *p* | *β* | 95% CI | *p* | *β* | 95% CI | *p* | *Exp(β)* | 95% CI | *P* |
| Group |  |  |  |  |  |  |  |  |  |  |  |  |
| TMS | 0.00 | Reference | - | 0.00 | Reference | - | 0.00 | Reference | - | 0.00 | Reference | - |
| IM | 0.97 | 0.62 to 1.52 | 0.895 | 0.12 | -1.13 to 1.36 | 0.852 | 4.39 | 2.01 to 6.76 | < 0.001 | 1.17 | 0.79 to 1.73 | 0.442 |
| Day |  |  |  |  |  |  |  |  |  |  |  |  |
| Day 0 | 0.00 | Reference | - | 0.00 | Reference | - | 0.00 | Reference | - | - | - | - |
| Day 1 | 2.66 | 1.97 to 3.59 | < 0.001 | -0.82 | -1.90 to 0.26 | 0.136 | -0.02 | -0.80 to 0.80 | 0.960 | 0.00 | Reference | - |
| Day 2 | 2.61 | 1.93 to 3.52 | < 0.001 | -1.16 | -2.34 to 0.02 | 0.053 | -0.54 | -1.37 to 0.29 | 0.200 | 1.20 | 0.99 to 1.45 | 0.071 |
| Day 3 | 1.27 | 0.90 to 1.79 | 0.173 | -2.11 | -3.44 to -0.78 | 0.002 | -0.78 | -1.68 to 0.12 | 0.091 | 0.64 | 0.49 to 0.82 | 0.001 |
| Day 4 | 0.69 | 0.46 to 1.02 | 0.065 | -2.64 | -4.15 to -1.12 | 0.001 | -0.37 | -1.36 to 0.63 | 0.468 | 0.36 | 0.24 to 0.56 | < 0.001 |
| Day 5 | 0.54 | 0.35 to 0.83 | 0.006 | -2.91 | -4.64 to -1.17 | 0.001 | -0.71 | -1.82 to 0.40 | 0.209 | 0.18 | 0.09 to 0.38 | < 0.001 |
| Day 6 | 0.34 | 0.20 to 0.58 | < 0.001 | -3.28 | -5.24 to -1.31 | 0.001 | -1.25 | -2.48 to -0.02 | 0.047 | 0.18 | 0.10 to 0.34 | < 0.001 |
| Day 7 | 0.26 | 0.14 to 0.47 | < 0.001 | -4.04 | -6.26 to -1.81 | < 0.001 | -0.61 | -1.99 to 0.76 | 0.383 | 0.08 | 0.03 to 0.19 | < 0.001 |
| Baseline | 1.24 | 1.10 to 1.40 | < 0.001 | 0.94 | 0.87 to 1.01 | < 0.001 | 0.69 | 0.52 to 0.85 | < 0.001 | - | - | - |
| Group by Day |  |  |  |  |  |  |  |  |  |  |  |  |
| IM x Day 2 |  |  |  |  |  |  |  |  |  | 0.68 | 0.49 to 0.95 | 0.023 |
| IM x Day 3 |  |  |  |  |  |  |  |  |  | 0.78 | 0.49 to 1.24 | 0.296 |
| IM x Day 4 |  |  |  |  |  |  |  |  |  | 0.70 | 0.36 to 1.37 | 0.299 |
| IM x Day 5 |  |  |  |  |  |  |  |  |  | 1.25 | 0.43 to 3.67 | 0.684 |
| IM x Day 6 |  |  |  |  |  |  |  |  |  | 0.73 | 0.24 to 2.21 | 0.580 |
| IM x Day 7 |  |  |  |  |  |  |  |  |  | 1.08 | 0.30 to 3.87 | 0.908 |

*Exp(β):* The coefficients and 95% CI for movement evoked pain intensity and daily average pain are exponentiated and represent a proportional change on the outcome measure for a 1 unit increase in the predictor/independent variable; *β:* The coefficients and 95 % CI for PCS and TSK represent a mean change in the outcome for a 1 unit change in the predictor/independent variable. TSM: Temporary Sensitisation Model; IM: Injury Model; PCS: Pain Catastrophising Scale; TSK: Tampa Scale of Kinesiophobia; 95% CI: 95% confidence interval.
